# Improved outcomes of older patients with acute and displaced proximal humerus fractures treated with window bone ingrowth fracture-specific stem reverse shoulder arthroplasty

**DOI:** 10.1186/s12877-023-04210-8

**Published:** 2023-09-12

**Authors:** Rui Claro, Ana Ribau, Hélder Fonte, Tiago Amorim-Barbosa, Luís Henrique Barros, Nuno Sevivas

**Affiliations:** 1 Department of Orthopaedics, Centro Hospitalar Universitário de Santo António, Porto, Portugal; 2 Department of Orthopaedics, Shoulder Unit, Centro Hospitalar Universitário de Santo António, Porto, 4099-001 Portugal; 3 Instituto de Ciências Biomédicas Abel Salazar da Universidade Do Porto (ICBAS-UP), Porto, Portugal; 4 Department of Orthopaedics, Hospital das Forças Armadas – Pólo Porto, Porto, Portugal; 5 Department of Orthopaedics, Centro Hospitalar Hospitalar Do Médio Ave, Vila Nova de Famalicão, Portugal; 6 Life and Health Sciences Research Institute (ICVS), School of Medicine, University of Minho, Campus de Gualtar, Braga, Portugal; 7 ICVS/3B’s - PT Government Associate Laboratory, Braga/Guimarães, Portugal; 8 Grupo Trofa Saúde, Trofa, Portugal

**Keywords:** Proximal humerus fractures, Reverse shoulder arthroplasty, Older people, Stem design, Tuberosity, Fixation technique

## Abstract

**Background:**

The optimal treatment of displaced proximal humerus fractures (PHFs) in the older people population remains controversial. Reverse shoulder arthroplasty (RSA) is a popular surgical treatment option that provides improved and reproducible results. However, the relevance of fracture-specific stem designs for RSA to improve tuberosity consolidation and shoulder function remains debatable.

**Methods:**

This study included all patients 70 years or older with acute and displaced PHFs primarily treated with RSA at a single institution in Portugal, between January 2010 and December 2019 who participated in a minimum follow-up of 2 years.

**Results:**

A total of 112 patients (15 men and 97 women) with a median clinical follow-up of 52 months were included. The mean age at the time of fracture was 78.6 years. All fractures were classified as Neer types 3 and 4 (*n* = 50 and *n* = 62, respectively). A window bone ingrowth fracture-specific stem was used for 86 patients, and a conventional humeral stem was used for 26 patients. Regarding the tuberosity fixation technique, 76 tuberosities were attached using technique A (according to Boileau's principles), 36 tuberosities were attached using technique B (not following Boileau's principles) and 11cases were classified as technique C (if fixation was not possible). The overall survival rate during the 2-year follow-up was 88.2%; however, this decreased to 79% at 5 years. Only three patients had complications (two infections and one dislocation) requiring revision surgery. In the multivariable analysis, the tuberosity fixation technique (*P* = 0.012) and tuberosity anatomical consolidation (*P* < 0.001) were associated with improved Constant scores (median Constant Score 62.67 (technique A), 55.32 (technique B), 49.70 (technique C). Fracture-specific humeral implants (*P* = 0.051), the tuberosity fixation technique (*P* = 0.041), tuberosity anatomical consolidation (*P* < 0.001), and dementia influenced the achievement of functional mobility (*P* = 0.014). Tuberosity anatomic consolidation was positively associated with bone ingrowth fracture-specific humeral implants (*P* < 0.01) and a strong tuberosity fixation technique (*P* < 0.01).

**Conclusion:**

RSA is used for complex and displaced fractures of the proximal humerus in older patients. Dementia was negatively correlated with functional outcomes. A window bone ingrowth fracture-specific stem combined with strong tuberosity fixation can yield better clinical and radiological results.

**Level of evidence:**

Level II; prospective comparative study; treatment study.

## Introduction

Proximal humeral fractures (PHFs) are a common fragility fractures in older patients that occur with low-energy trauma mechanisms and are sentinel markers of poor bone health and a general decline in health [1–5]. At 1 year after a PHF, the mortality rate is 10% regardless of treatment [6]. Patients who do not live alone, do not participate in recreational activities, and cannot independently perform activities of daily living are at significantly higher risk for poor outcomes [7].

Several factors, such as patient anatomy, fracture pattern, pre-injury functional status, medical comorbidities, and surgeon skill level, make it difficult to successfully regain a functional shoulder. Currently, 67% to 85% of PHFs are treated non-operatively [8, 9], and the best treatment for older patients remains controversial. Nevertheless, the rate of surgical intervention is increasing [8, 10]. The use of open reduction and internal fixation has remained stable in recent years, that of hemiarthroplasty has decreased, and that of reverse shoulder arthroplasty (RSA) has increased [11].

RSA has become the preferred prosthetic treatment option for complex PHFs of older patients requiring surgical treatment because it is less dependent on tuberosity healing for the maintenance of function and stability. Multiple studies have demonstrated improved functional outcomes of patients who underwent RSA compared to those who underwent hemiarthroplasty [12]. However, achieving tuberosity healing is desirable after RSA because patients with tuberosity healing may have improved functional outcomes [13].

Tuberosity healing after RSA for fractures depends on several factors, including the repair technique, prosthesis design, patient comorbidities, and postoperative rehabilitation. Implant variables such as an inlay versus an onlay humeral implant, glenosphere offset, humeral component neck–shaft angle, and the use of a humeral implant with a “window” for autologous bone graft change the postoperative position of the tuberosities and consequent tension and healing of the tuberosity repair [9, 11–14]. However, few studies have compared the effects of changing these factors.

We evaluated patients 70 years or older treated primarily with RSA after displaced PHFs and identified mortality, morbidity, complications, reoperations, and functional outcomes. We also evaluated the effect of a fracture-specific RSA design with different tuberosity fixation methods on the implant survival rate and safety of the procedure.

We hypothesised that RSA with a fracture-specific design using a “window” stem and with a strong tuberosity fixation would improve anatomical tuberosity repair and consolidation, thereby providing better functional results.

## Materials and methods

A retrospective analysis of the surgical activity of a single orthopaedic department between January 1, 2010 and December 31, 2019 was performed. Data of all surgeries performed for PHFs during the study period were prospectively collected. The inclusion criteria were patients 70 years or older who underwent RSA as a primary option for displaced PHFs (displaced Neer types 3 and 4) and a minimum of 2 years of follow-up. The decision for RSA was based on pre-operative consensus among two independent surgeons who agreed that achieving a satisfactory reduction intra-operatively was not feasible. The exclusion criteria were pathologic fractures and time to surgery of more than 6 weeks. Before inclusion, approval from the ethical committees was obtained as required by local regulations, and informed consent was obtained from all participants.

**Fig. 1:**
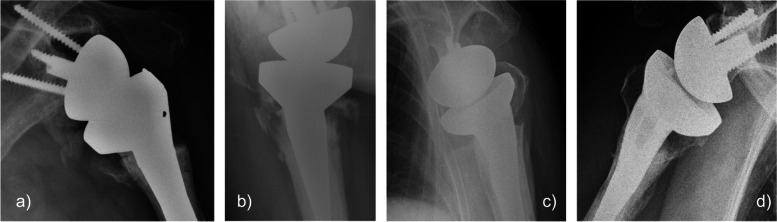
Radiographic evaluation of tuberosity healing. Four different classifications: **a** resorption; **b** non-union; **c** malunion; and **d** anatomical healing

A preoperative assessment was performed using radiography and computed tomography for all patients. Follow-up was conducted by a senior surgeon at 2 weeks, 1 month, 3 months, 6 months, and 1 year, and then annually thereafter. Plain radiography (anteroposterior views in neutral rotation, external rotation, and internal rotation; lateral scapula shoulder or Y view; Velpeau view) was performed during the follow-up consultations at 4 weeks, 3 months, 6 months, and 1 year, and then annually thereafter. For all patients, the mean cortical thickness was measured using Tingart’s method at the time of the first X-ray examination [15]. Tuberosity healing was classified as anatomical healing, malunion and non-union, and resorption according to the classification by Imiolczyk et al. (Fig. 1) [14]. Furthermore, heterotopic ossification (a modified Brooker classification system for the hip was used for its grading) [16], implant loosening (was evaluated according to the classification of Gruen et al., [17] adapted to the shoulder), and scapular notching (was evaluated according to the Nerot-Sirveaux classification) [18, 19] were included in the radiological evaluation.

Demographic data were collected from the clinical records and included sex, age at the time of fracture, American Society of Anaesthesiology (ASA) score, comorbidities, Charlson comorbidity index [20], associated injuries, time to surgery, Constant shoulder score [21], visual analogue scale score, functional mobility based on the study by Namdari et al. [22] (forward elevation > 115°, > 40° of extension, abduction at least 120°, > 105° of cross-body adduction, external rotation with the arm 90°, abduction > 50°, and internal rotation with the arm at the side > 95°), return to activities of daily living (short questionnaire based on the International Physical Activity Questionnaire Short Form [23] completed by the surgeon during the follow-up consultations comparing the level of activity before and after the fracture), mortality rate, complications (tuberosity non-union, tuberosity malunion, heterotopic ossification, glenoid erosion, notching, loosening, re-operation, infection, and instability of the implant), and mortality.

### Surgical technique

Surgery was performed by a senior shoulder surgeon using a deltopectoral approach for all patients. The long head of the biceps tendon was tenodesed to the pectoralis major whenever it was present. Closed suction drainage was used and removed within 24 h for all patients.

**Fig. 2:**
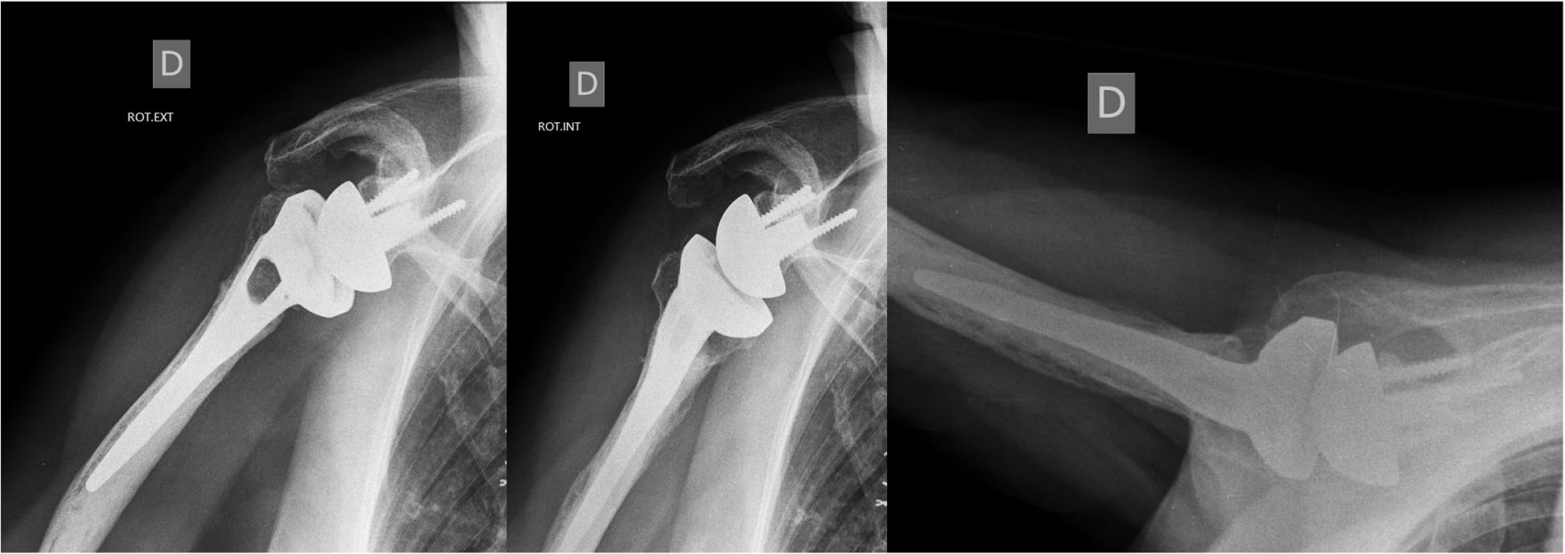
X-ray evaluation of the fracture-specific humeral stem (Aequalis Reversed-Fracture implant; Tornier, Memphis, TN, USA)

**Fig. 3:**
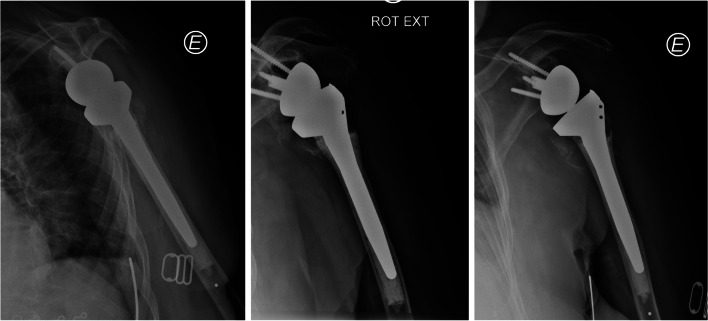
X-ray evaluation of the conventional humeral stem (Aequalis Reversed II implant; Tornier, Memphis, TN, USA)

All humeral implants were cemented and assembled using a specific instrument to control the height (sliding ruler). Retroversion was set at 20° by aligning the version rod to the forearm. The humeral stem diameter corresponded to the diameter of the last broach used. The humeral implant selected for the study was an implant with a specific stem for the fracture (Aequalis reversed-fracture implant; Tornier, Memphis, TN, USA). This fracture-specific stem is a monobloc stem with a hydroxyapatite coating and metaphyseal window for bone augmentation to encourage fracture healing, especially tuberosity healing (Fig. 2). For these cases, a specially shaped cancellous bone graft was harvested from the fractured head using appropriate instruments and placed into a designated window in the prosthesis. Whenever this implant was not available because of distribution issues, a similar humeral stem without a window compatible with the same glenoid implant was used (Aequalis Reversed II; Tornier, Memphis, TN, USA). This conventional stem consists of two components: a cobalt-chrome diaphyseal stem and a metaphysis with an anti-rotation design (Fig. 3).

After retraction of the tuberosities, the glenoid surface was reamed with a cannulated reamer inserted over a guidewire, and a central hole for the glenoid peg was drilled at 0° of inferior tilt. The baseplate was always impacted and secured in place with four screws (two compression and two locking screws). A standard 36-mm-diameter glenosphere was used for all patients.

**Fig. 4:**
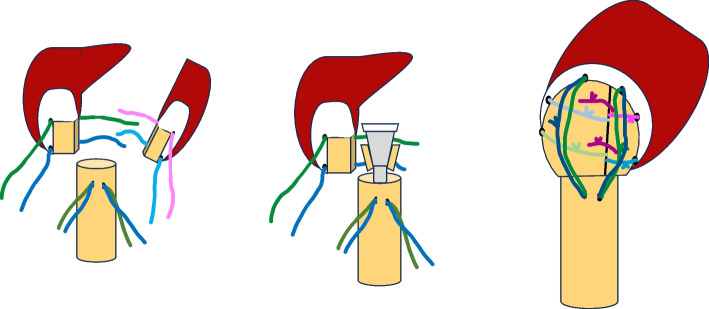
Schematic figures with technique A

**Fig. 5:**
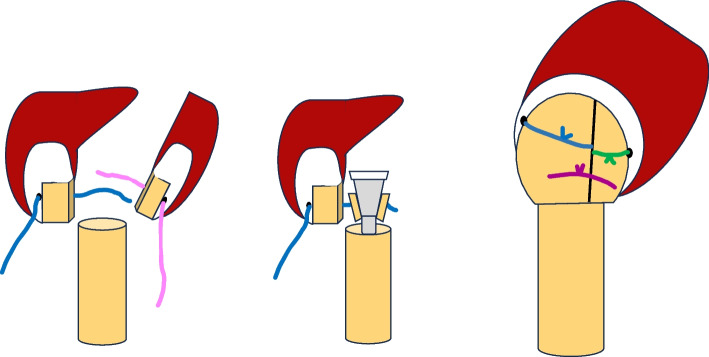
Schematic figures with technique B

Regarding fixation of the tuberosities, reinsertion was always attempted. When intraoperative fixation was possible, two of the four senior surgeons [RC and LB] always used technique A (Fig. 4) according to Boileau’s principles [24]. This technique was defined as a minimum of two no. 5 Ethibond sutures (Ethicon Inc., Somerville, NJ, USA) passing through the tendon–bone interface of the tuberosities and embracing the humeral implant (horizontal cerclage sutures), two vertical no. 5 Ethibond sutures (vertical tension-band sutures), and a minimum of two mo. 5 Ethibond sutures attaching both tuberosities. The other two senior surgeons [JL and JR] always used technique B (Fig. 5), whereby only one suture passed through the tuberosities and embraced the humeral implant without vertical sutures; only one intertuberosity suture could be used. The technique was classified as technique C if fixation was not possible because of poor bone quality, absence of tuberosity, or absence of tendons (chronic ruptures and significant tendon retraction). All surgeries were performed successively according to the rotation of the emergency department schedule. No surgery was scheduled for a particular surgeon. No patient was assigned to a specific surgeon.

After surgery, the shoulder was immobilised with a brace for 4 weeks. Active wrist and elbow movements were immediately allowed. Passive mobilisation of the shoulder was allowed at 2 weeks and active mobilisation was allowed at 6 weeks postoperatively. All patients underwent the same rehabilitation protocol.

The demographic data were presented by descriptive statistic. For categorical variables, the number of cases and percentages were presented, and the chi-square test or Fisher's exact test were used to compare proportions between groups. Continuous variables were presented as means and standard deviations (SD). The Shapiro–Wilk test was used to assess the distribution of the variables, and the t-test or Mann–Whitney U test (for non-normally distributed variables) were utilized to compare means between groups.

All statistical analyses were performed using IBM SPSS Statistics (version 24.0; New York, New York, USA). Statistical significance was set as a two-tailed P-value less than 0.05 and 95% confidence interval (CI) were reported. The multivariable analysis was performed including all variables that demonstrate a difference in the univariate analysis with *p* ≤ 0.2, through a binomial logistic regression.

## Results

Between January 1, 2010 and December 31, 2019, 653 patients with PHFs were included in the study. Of these patients, 420 underwent open reduction and osteosynthesis with a plate, 32 underwent nailing, 10 underwent hemiarthroplasty, 12 underwent surgery for pathological fractures, 45 patients younger than 70 years underwent RSA and 22 waited for more than 6 weeks before surgery was performed, were excluded. Finally, 112 met the inclusion criteria.

Of the 112 patients included (112 shoulders), 15 were men and 97 were women; the mean clinical follow-up was 52 months (SD, 27.9 months). The mean age at the time of fracture was 78.6 years (SD, 5.82 years). According to the Charlson comorbidity index, 41 patients had a score of 4 and 17 patients had a score ≥ 6. Sixty-three patients (56.25%) had an ASA score of II, 47 (41.96%) had an ASA score of III, and two (1.79%) had an ASA score of IV. The mean time from trauma to surgery was 6.9 days (SD, 7.84 days).

All fractures were classified as Neer types 3 and 4. Fifty patients (45%) had three-part fractures and 62 patients (55%) had four-part fractures. The mean cortical thickness was 2.83 mm (SD, 0.672 mm). The Aequalis Reversed-Fracture stem (Tornier, Memphis, TN, USA) humeral implant was used for 86 patients (76.8%). The Aequalis Reversed II humeral stem (Tornier, Memphis, TN, USA) was implanted in 26 patients (23.2%). Regarding the tuberosity fixation technique, the tuberosities were attached using technique A for 76 patients (67.9%), technique B for 25 patients (22.3%) and technique C for 11 patients (9.8%) (Tables 1 and 2, respectively). Of all patients who received a fracture-specific humeral implant, 59 patients (69%) underwent technique A fixation, 19 patients (22%) underwent technique B fixation, and 8 patients (9%) underwent technique C fixation. Of all patients who received the non-fracture humeral stem, 17 patients (65%) underwent technique A fixation, 6 patients (23%) underwent technique B fixation, and 3 patients (12%) underwent technique C fixation.

**Table 1:** Demographic characteristics and comorbidities

**Variable**	**n (%)**	**Mean (SD)**
**Sex**		
Male	15 (13.4)	
Female	97 (86.6)	
**Age (years)**		78.6 (5.82)
**ASA score**		
II	63 (56.25)	
III	47 (41,96)	
IV	2 (1.79)	
**Comorbidities**		
Diabetes mellitus	25 (22.3)	
Hypertension	71 (63.4)	
Heart failure	37 (33)	
Respiratory failure	19 (17)	
Renal failure	10 (8.9)	
History of stroke	11 (9.8)	
Obesity	34 (30.4)	
Dementia	10 (8.9	
**Charlson comorbidity index**		
2	6 (5.4)	
3	19 (17)	
4	41 (36.6)	
5	26 (23.2)	
≥ 6	17 (15.2)	

*ASA* American Society of Anesthesiology, *SD* Standard deviation

**Table 2:** Surgical characteristics

Variable	N (%)	Mean (SD)
Laterality		
Right	65 (57.1)	
Neer’s classification		
3	50 (44.6)	
4	62 (55.4)	
Mean cortical thickness (mm)		2.83 (0.672)
Implant		
ARF	86 (76.8)	
Other	26 (23.2)	
Tuberosity fixation technique		
Technique A	76 (67.9)	
Technique B	25 (22.3)	
Technique C	11 (9.8)	
Postoperative transfusion	15 (13.4)	
Time to surgery (days)		6.9 (7.84)
Length of postoperative hospital stay (days)		5.2 (10.31)
Follow-up (months)		52 (27.9)

*ARF* Aequalis Reversed II humeral stem, *SD* Standard deviation

**Fig. 6:**
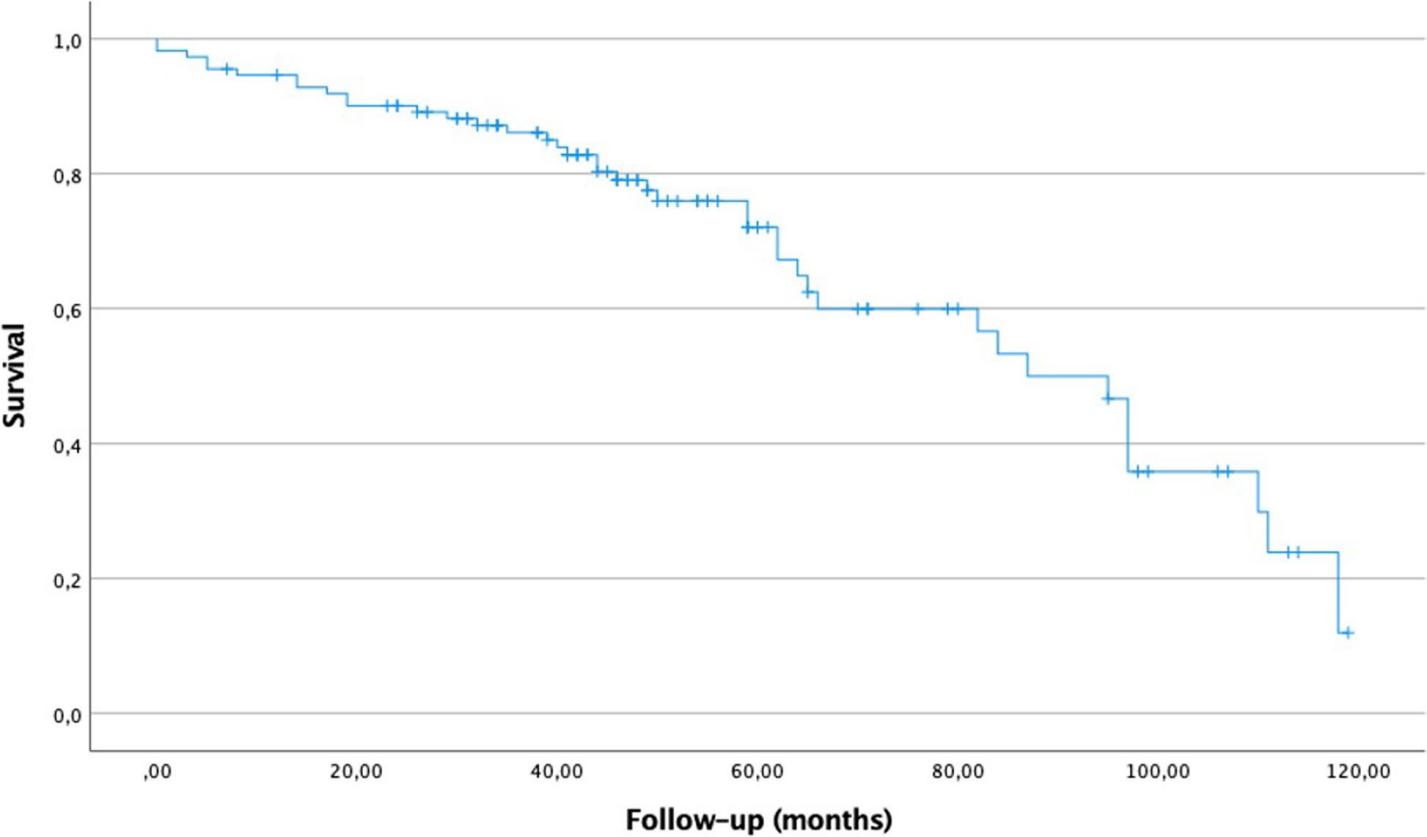
Overall survival

To assess survival rates, the need for revision surgery or patient death was considered a survival endpoint. The overall survival rate at 2 years of follow-up was 88.2%; however, it decreased to 79% at 5 years. Figure 6 shows the Kaplan–Meier survival curve.

Regarding functional outcomes, the majority of patients (73.2%) returned to their normal activities of daily living, and 45.5% of patients achieved functional mobility (Table 3). The mean visual analogue scale score was 3.6 (SD, 2.31).

**Table 3:** Postoperative outcomes

Variable	N (%)	Mean (SD)
**Complications**		
Infection	2 (1.8)	
Dislocation	1 (0.9)	
**Revision surgery**	3 (2.7)	
**Return to daily activities**	82 (73.2)	
**Functional mobility**	51 (45.5)	
**Constant score**		59.4 (10.40)
**VAS**		3.6 (2.31)

*SD* Standard deviation, *VAS* Visual analogue scale

Only three patients (2.7%) had complications (two infections [1.8%] and one dislocation [0.9%]) that required revision surgery (Table 3).

Of the initial 112 patients, 19 missed radiological appointments during the first 2 years of follow-up. Therefore, only 93 patients underwent shoulder radiograph evaluation during a minimum of 2 years of follow-up and were included in the subsequent analysis (Table 4).

**Table 4:** Radiological outcomes

Variable	N (%)
Tuberosity anatomic consolidation	58 (62%)
Tuberosity malunion	13 (14%)
Tuberosity nonunion	15 (16%)
Tuberosity resorption	7 (8%)
Scapular notching	26 (28%)
Heterotopic ossification	35 (38%)
Implant loosening	3 (3%)

In the multivariate analysis, the type of humeral implant (*p* = 0.051; 95% confidence interval [CI] 0.997–12.964, odds ratio (OR) = 3.595), tuberosity fixation technique (*p* = 0.041; 95% CI 0.179–0.963, OR = 0.415), tuberosity anatomical consolidation (*p* < 0.001; 95% CI 2.236–23.031, OR = 7.315), and dementia (*p* = 0.014; 95% CI 0.010–0.601, OR = 0.077 were the only factors that influenced the achievement of functional mobility.

Additionally, the tuberosity fixation technique (*p* < 0.01; 95% CI 5.387—0.686) and tuberosity anatomical consolidation (*p* < 0.01; 95% CI 10.471–16.969) were associated with an improved Constant score (Table 5).

**Table 5:**
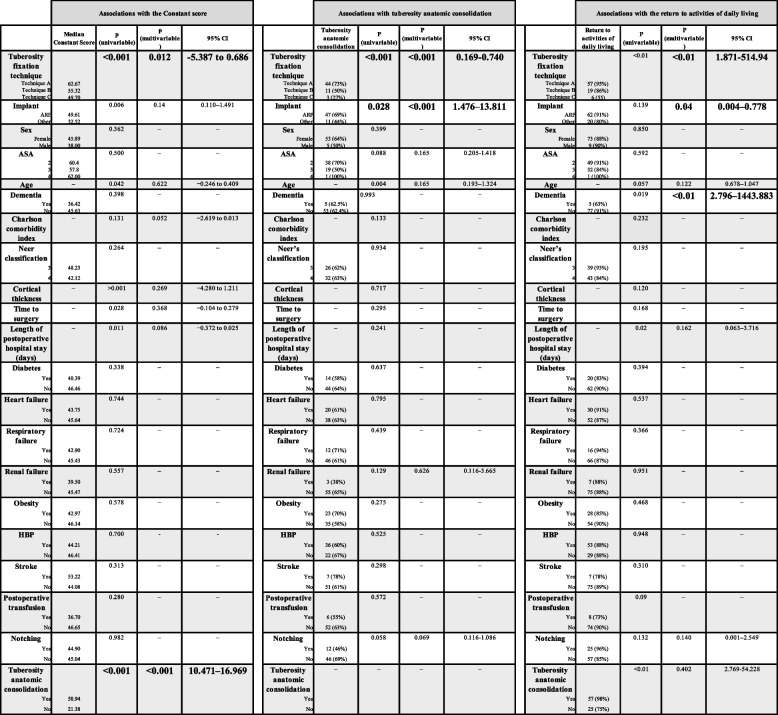
Associations with the constant score, with tuberosity anatomic consolidation, and with the return to activities of daily living

*ARF* Aequalis reverse fracture, *ASA *American Society of Anesthesiology, *CI *Confidence interval, *HBP* High blood pressure

Tuberosity anatomic consolidation was associated with the type of humeral implant (*p* < 0.001; 95% CI 1.476–13.811,) and fixation technique (*p* < 0.001; 95% CI 0.169–0.740). No associations with Neer’s fracture classification (*p* = 0.934, 95% CI 0.19- 1.33), time to surgery (*p* = 0.295 95% CI -13.11–4.61), or cortical thickness (*p* = 0.717, 95% CI 0.6–3.61) were observed between groups (Table 5).

The factors associated with the ability to return to performing activities of daily living were the type of implant (*p* = 0.044; 95% CI, 1.05–92.47), tuberosity fixation technique (*p* = 0.12; 95% CI 0.051–0.695), and dementia (*p* = 0.014; 95% CI 0.002–0.493) (Table 5).

## Discussion

To the best of our knowledge, this study is one of the few that evaluated a fracture-specific design using a “window” humeral stem and standard humeral stem for the treatment of acute and displaced PHFs in older patients. We compared different fixation techniques for tuberosities and their ability to improve anatomical consolidation and provide better functional results. Our results revealed that consolidation of the tuberosity in the anatomic position is associated with the use of a fracture-specific stem and strong fixation technique.

Although approximately 80% of PHFs are non-displaced or minimally displaced [25], and because non-operative treatment is successful [26], optimal treatment becomes less clear for more complex displaced fracture patterns, such as those that occur in the older people.

The poor bone quality of older patients causes complex fracture patterns that make open reduction and internal fixation impossible; when performed, open reduction and internal fixation result in a high rate of complications, with some series reporting a complication rate as high as 50% [27].

Tuberosity integration with RSA is not as important for function as it is with hemiarthroplasty; therefore, for older patients with osteoporotic bones, the use of RSA has some advantages, at least theoretically. If the tuberosities heal in an anatomical position, then, theoretically, RSA will be advantageous in terms of final functional outcomes. Moreover, eventually, the use of specific stems for fractures will be necessary to obtain anatomical healing of the tuberosities.

A recent meta-analysis comparing the fracture-specific stem design for hemiarthroplasty and RSA has reported favourable outcomes for fracture stems regardless of different rehabilitation protocols or tuberosity refixation techniques [28]. Different conclusions were presented by Imiolczyk et al. [14]; in their study, fracture stem designs with an open metaphyseal window for bone ingrowth did not improve tuberosity healing.

Tuberosity healing rates of 65% to 84% have been described in the literature, and our study revealed a healing rate of 76% (62% anatomical consolidation and 14% malunion) [24, 29–31]. Our study revealed that consolidation of tuberosity in the anatomic position was associated with the use of a fracture-specific stem (*P* < 0.01) and the fixation technique used (*P* < 0.01), but that there was no association with Neer’s classification (*P* = 0.934), time to surgery (*P* = 0.295), or cortical thickness (*P* = 0.717).

Our data also suggested that the tuberosity fixation technique and anatomical consolidation of the tuberosity are associated with improvements in the Constant score, thus corroborating the findings of Grubhofer et al. [31], Boileau et al. [24], Barros et al. [32], and Jain et al. [13], who reported that patients with tuberosity healing had improved functional outcomes and subjective function.

Hence, in order to promote optimal anatomical consolidation and improve functional recovery of the affected upper limb, it is advisable to prioritize the use of a fracture-specific stem with a window for bone ingrowth. Additionally, when feasible during the surgical procedure, the implementation of a robust fixation technique, as outlined by Boileau et al. [24], should be considered. This combined approach offers the potential to mitigate the risk of tuberosity mal-union or non-union, which holds particular significance in the context of this age group.

RSA allowed the return to activities of daily living for 73.2% of patients and functional mobility of the shoulder for almost half (45.5%) of the patients studied (45.5%); however, dementia was negatively correlated with the ability to return to activities of daily living. These factors, particularly for the older population who often live alone and need to recover quickly, suggest that RSA should be the preferred surgical option because of its good results when dealing with displaced PHFs. Nonetheless, older people should always undergo rigorous preoperative assessments. Their treatment should be optimised according to the surgical risk profile before surgery, and the option for surgical treatment should always be evaluated on a case-by-case basis.

This study had some limitations. First, the relatively small number of patients and relatively high rate of loss to radiological follow-up (16.96%) may have hindered the detection of small differences between groups. This was because of the age and morbidities of the patient cohort and was typical of other cohort studies of RSA for PHFs, which reported dropout rates of approximately 29% to 34% [24, 29, 31]. Second, only a two-dimensional radiographic evaluation of tuberosity healing was performed. Three-dimensional computed tomography may provide a more accurate evaluation of tuberosity integration and positioning, but at the expense of higher radiation exposure. Third, the clinical evaluation at follow-up was performed by the senior surgeon who performed the surgery. Fourth, a relatively small number of patients were treated with a conventional humeral stem. A randomized, comparative, control study should be performed to avoid these limitations.

Nevertheless, the strengths of our study include the long follow-up period, the use of the same glenoid reconstruction, and the use of same neck–shaft angle of the humeral stem (Grammont type, 155°). Furthermore, all surgeries were performed by senior orthopaedic surgeons, and the same rehabilitation protocol was used for all patients to investigate the effects of different stem designs and different tuberosity fixation techniques. Studies with larger patient cohorts are necessary to clarify all factors that can help surgeons select the optimal procedure.

## Conclusion

RSA is often used for complex and displaced fractures of the proximal humerus in older patients. Dementia was negatively correlated with functional outcomes. Better clinical and radiological results can be achieved with a window bone ingrowth fracture-specific stem combined with strong tuberosity fixation.

## Data Availability

The datasets used and/or analyzed during the current study available from the corresponding author on reasonable request. The data are not publicly available due to ethical restrictions.

## References

[1] Rotman D, Giladi O, Senderey AB, Dallich A, Dolkart O, Kadar A, Maman E, Chechik O (2018). Mortality after complex displaced proximal humerus fractures in elderly patients: conservative versus operative treatment with reverse total shoulder arthroplasty. Geriatr Orthop Surg Rehabil.

[2] Verdano MA, Aliani D, Galavotti C, Maroun C, Vaienti E, Ceccarelli F (2018). Grammont versus lateralizing reverse shoulder arthroplasty for proximal humerus fracture: functional and radiographic outcomes. Musculoskelet Surg.

[3] Maugendre E, Gadisseux B, Chantelot C, Clavert P, Ramdane N, Werthel JD, Boileau P, Sofcot (2019). Epidemiology and mortality in older patients treated by reverse shoulder arthroplasty for displaced proximal humerus fractures. Orthop Traumatol Surg Res.

[4] Fitschen-Oestern S, Behrendt P, Martens E, Finn J, Schiegnitz J, Borzikowsky C, Seekamp A, Weuster M, Lippross S (2020). Reversed shoulder arthroplasty for the treatment of proximal humerus fracture in the elderly. J Orthop.

[5] Court-Brown CM, Caesar B (2006). Epidemiology of adult fractures: a review. Injury.

[6] Kim SH, Szabo RM, Marder RA (2012). Epidemiology of humerus fractures in the United States: nationwide emergency department sample, 2008. Arthritis Care Res (Hoboken).

[7] Clark NJ, Samuelsen BT, Alentorn-Geli E, Assenmacher AT, Cofield RH, Sperling JW, Sanchez-Sotelo J (2019). Primary reverse shoulder arthroplasty in patients older than 80 years of age: survival and outcomes. Bone Joint J.

[8] Brauer CA, Coca-Perraillon M, Cutler DM, Rosen AB (2009). Incidence and mortality of hip fractures in the United States. JAMA.

[9] Dhaliwal K, Shahid ZY, Choudhry B, Zhao C (2020). The Role of Reverse Shoulder Arthroplasty in Elderly Trauma: A Systematic Review. Cureus..

[10] Bufquin T, Hersan A, Hubert L, Massin P (2007). Reverse shoulder arthroplasty for the treatment of three- and four-part fractures of the proximal humerus in the elderly: a prospective review of 43 cases with a short-term follow-up. J Bone Joint Surg Br.

[11] Kelly BJ, Myeroff CM (2020). Reverse Shoulder Arthroplasty for Proximal Humerus Fracture. Curr Rev Musculoskelet Med.

[12] O'Sullivan J, Ladermann A, Parsons BO, Werner B, Steinbeck J, Tokish JM, Denard PJ (2020). A systematic review of tuberosity healing and outcomes following reverse shoulder arthroplasty for fracture according to humeral inclination of the prosthesis. J Shoulder Elbow Surg.

[13] Jain NP, Mannan SS, Dharmarajan R, Rangan A (2019). Tuberosity healing after reverse shoulder arthroplasty for complex proximal humeral fractures in elderly patients-does it improve outcomes? A systematic review and meta-analysis. J Shoulder Elbow Surg.

[14] Imiolczyk JP, Moroder P, Scheibel M (2021). Fracture-Specific and Conventional Stem Designs in Reverse Shoulder Arthroplasty for Acute Proximal Humerus Fractures-A Retrospective, Observational Study. J Clin Med..

[15] Tingart MJ, Apreleva M, von Stechow D, Zurakowski D, Warner JJ (2003). The cortical thickness of the proximal humeral diaphysis predicts bone mineral density of the proximal humerus. J Bone Joint Surg Br.

[16] Della Valle AG, Ruzo PS, Pavone V, Tolo E, Mintz DN, Salvati EA (2002). Heterotopic ossification after total hip arthroplasty. J Arthroplasty.

[17] Gruen TA, McNeice GM, Amstutz HC (1979). "Modes of failure" of cemented stem-type femoral components: a radiographic analysis of loosening. Clin Orthop Relat Res..

[18] Young BL, Cantrell CK, Hamid N (2018). Classifications in Brief: The Nerot-Sirveaux Classification for Scapular Notching. Clin Orthop Relat Res.

[19] Sirveaux F, Favard L, Oudet D, Huquet D, Walch G, Mole D (2004). Grammont inverted total shoulder arthroplasty in the treatment of glenohumeral osteoarthritis with massive rupture of the cuff. J Bone Joint Surg Br.

[20] Charlson ME, Pompei P, Ales KL, MacKenzie CR (1987). A new method of classifying prognostic comorbidity in longitudinal studies: development and validation. J Chronic Dis.

[21] Constant CR, Murley AH (1987). A clinical method of functional assessment of the shoulder. Clin Orthop Relat Res..

[22] Namdari S, Yagnik G, Ebaugh DD, Nagda S, Ramsey ML, Williams GR, Mehta S (2012). Defining functional shoulder range of motion for activities of daily living. J Shoulder Elbow Surg.

[23] Sember V, Meh K, Soric M, Starc G, Rocha P, Jurak G (2020). Validity and Reliability of International Physical Activity Questionnaires for Adults across EU Countries: Systematic Review and Meta Analysis. Int J Environ Res Public Health..

[24] Boileau P, Alta TD, Decroocq L, Sirveaux F, Clavert P, Favard L, Chelli M (2019). Reverse shoulder arthroplasty for acute fractures in the elderly: is it worth reattaching the tuberosities?. J Shoulder Elbow Surg.

[25] Villodre-Jimenez J, Estrems-Diaz V, Diranzo-Garcia J, Bru-Pomer A (2017). Reverse shoulder arthroplasty in 3 and 4 part proximal humeral fractures in patients aged more than 65 years: Results and complications. Rev Esp Cir Ortop Traumatol.

[26] Gaebler C, McQueen MM, Court-Brown CM (2003). Minimally displaced proximal humeral fractures: epidemiology and outcome in 507 cases. Acta Orthop Scand.

[27] Owsley KC, Gorczyca JT (2008). Fracture displacement and screw cutout after open reduction and locked plate fixation of proximal humeral fractures [corrected]. J Bone Joint Surg Am.

[28] Onggo JR, Nambiar M, Onggo JD, Hau R, Pennington R, Wang KK (2021). Improved functional outcome and tuberosity healing in patients treated with fracture stems than nonfracture stems during shoulder arthroplasty for proximal humeral fracture: a meta-analysis and systematic review. J Shoulder Elbow Surg.

[29] Schmalzl J, Jessen M, Sadler N, Lehmann LJ, Gerhardt C (2020). High tuberosity healing rate associated with better functional outcome following primary reverse shoulder arthroplasty for proximal humeral fractures with a 135 degrees prosthesis. BMC Musculoskelet Disord.

[30] Simovitch RW, Roche CP, Jones RB, Routman HD, Marczuk Y, Wright TW, Zuckerman JD (2019). Effect of tuberosity healing on clinical outcomes in elderly patients treated with a reverse shoulder arthroplasty for 3- and 4-part proximal Humerus fractures. J Orthop Trauma.

[31] Grubhofer F, Wieser K, Meyer DC, Catanzaro S, Beeler S, Riede U, Gerber C (2016). Reverse total shoulder arthroplasty for acute head-splitting, 3- and 4-part fractures of the proximal humerus in the elderly. J Shoulder Elbow Surg.

[32] Barros LH, Figueiredo S, Marques M, Rodrigues C, Ramos J, Claro R (2020). Tuberosity healing after reverse shoulder arthroplasty for proximal Humerus fractures: is there clinical improvement?. Rev Bras Ortop (Sao Paulo).

